# Bilaminar Technique with Coronally Advanced Flap and Cryopreserved Human Amniotic Membrane in the Treatment of Gingival Recessions

**DOI:** 10.1155/2020/7827092

**Published:** 2020-08-26

**Authors:** Mario Martelloni, Pietro Boccaletto, Giulia Montagner, Diletta Trojan, Roberta Abate

**Affiliations:** ^1^Private Dental Practice, Via Genzano 191, 00179 Roma, Italy; ^2^Fondazione Banca dei Tessuti di Treviso Onlus, Piazzale Ospedale 1, 31100 Treviso, Italy; ^3^Private Dental Practice, Via Pietrasecca 2, 00132 Roma, Italy

## Abstract

Gingival recessions are usually treated with surgical therapies which involve the use of connective tissue autograft in order to thicken the gingival tissue. This has an important biological repercussion on patients since they will have surgical wounds in two different oral cavity sites. In this case report, we offer an alternative technique for the treatment of a Miller class I gingival recession. A 40-year-old female patient has been treated with a combination of bilaminar and coronally advance flap techniques to cover a Miller class I gingival recession with addition of cryopreserved human amniotic membrane. The human amniotic membrane has been employed to thicken the keratinized tissue. The human amniotic membrane in combination with bilaminar and coronally advance flap techniques has produced aesthetically and functionally satisfying results, covering completely the gingival recession and restoring the natural colour and thickness of the gingiva. Bilaminar technique with coronally advanced flap and cryopreserved human amniotic membrane is a valid alternative in treating Miller class I and II gingival recessions and reduce the biological morbidity of a double intervention.

## 1. Introduction

Gingival recession (GR) is defined as the apical displacement of the gingival margin from its physiological position to the cementoenamel junction (CEJ), with pathological exposure of the root surface [1]. It is often associated with the growth of root caries, dentin hypersensitivity, and blemish [2]. Epidemiological studies demonstrate that GR lesions are very frequent both in populations that keep good domiciliary oral hygiene and those with an insufficient one [3]. GR is also evenly spread through different ethnic groups [4]. GR are frequently the result of traumatic events, such as the inappropriate use of the toothbrush, occlusal trauma [5], or the anatomic-pathologic result of periodontitis. These lesions can also be related with predisposing factors like thin phenotype, decreased alveolar bone crest thickness, dehiscence, and “short frenulum” [5]. Mucogingival surgery is rich in techniques finalized to the resolution of GR. Coronally advanced flap (CAF), connective epithelium free graft, and connective subepithelium and tunnelling are just few of the most used techniques; although, numerous systematic reviews have shown that the subepithelial connective tissue graft is the gold standard procedure to obtain positive results in the cases of root covering. Despite the fact that autogenous connective tissue graft is considered the gold standard material for treating gingival recession [6], there is an important drawback due to patient morbidity to consider. Additionally, different soft tissue grafts have been used (such as collagen matrix, acellular dermal matrix, and human amniotic membrane). The predictability of this procedure is influenced by a series of factors including patient's habits, among which the most important is the smoking [7], the entity of the lesion, the tension of the strip, and the defect itself. Once identified the aetiology of the recession, it is solely the talent and the rationale of the surgeon to identify the most suitable technique to treat each single case, trying to reduce as much as possible the morbidity, but at the same time guaranteeing the full coverage of the GR with functional, aesthetically excellent results and positive long-term prognosis.

This case report describes a case of Miller class I gingival recession treated with the bilaminar technique with CAF and human amniotic membrane (HAM). HAM or amnion is a thin membrane on the inner side of the placenta. This tissue is widely used in different clinical applications such as treatment of skin burns and prevention of tissue adhesion in surgery of the head, neck, abdomen, genitourinary tract and larynx, ocular surface reconstruction, and wound healing [8] for its properties. HAM has various characteristics; it has antimicrobial [9] and anti-inflammatory functions [10], combined with immunological, antiangiogenic properties [8]. Moreover, it promotes epithelialization [11] making it ideal in the oral and periodontal field [12]. CAF is a root-coverage technique extensively used because of its high success rate, and it has been used with foetal membranes to obtain a good clinical outcome [13].

## 2. Case Report

**Figure 1:**
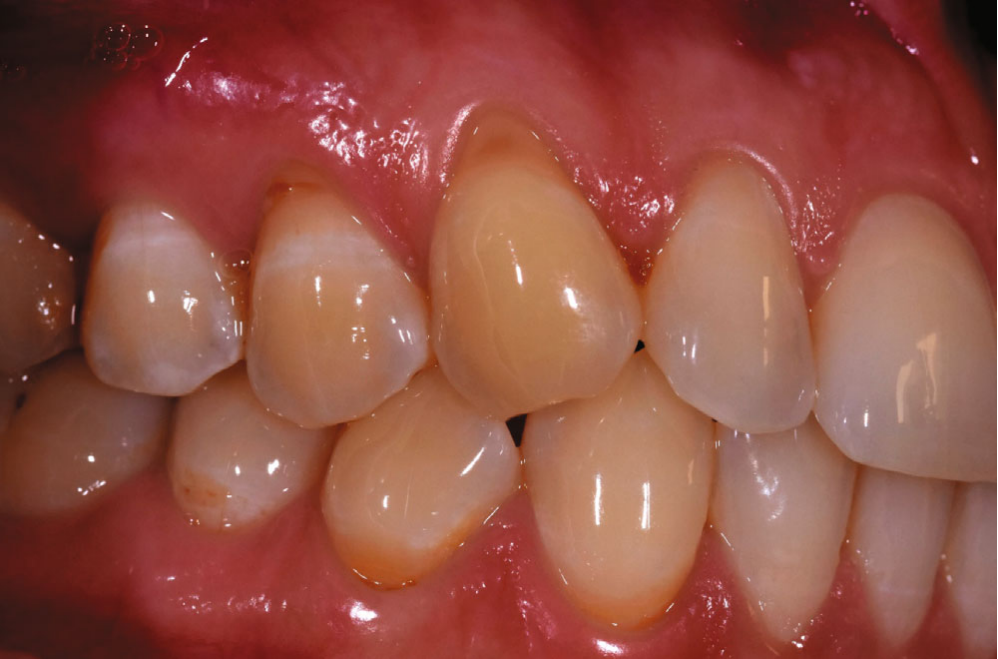
Photo of the patient taken at the moment of GR diagnosis. GR on tooth 1.3 and 1.4 was recognized by the displacement of the gingival margin from its physiological position.

**Figure 2:**
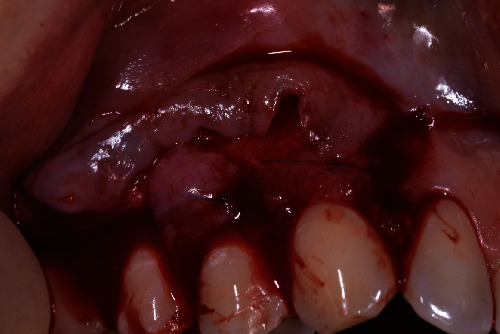
Folded HAM placed at contact with the periosteum in the surgical site.

**Figure 3:**
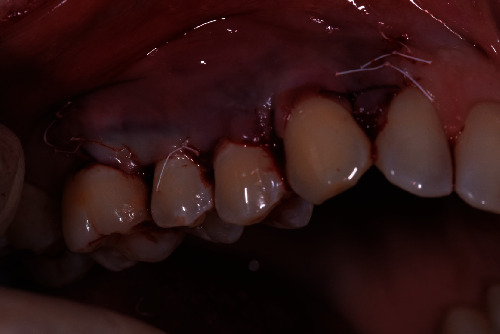
HAM stitched above the gingival recession site.

**Figure 4:**
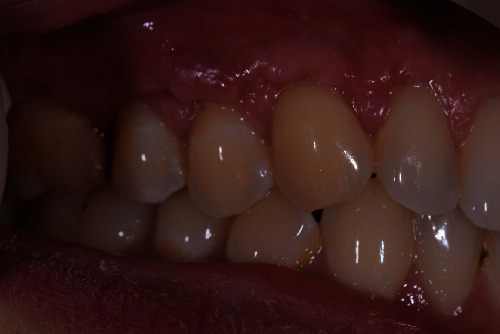
Photo taken 15 days postintervention after stitches removal.

**Figure 5:**
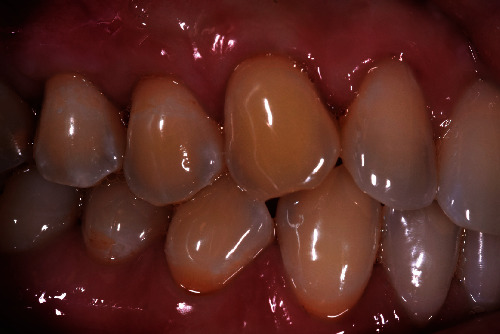
The healed site presents itself without gingival recessions and with restored shape and colour of the gingiva.

A 40-year-old female patient, nonsmoker with good oral hygiene, turned to the private practice complaining of an aesthetic discomfort in the smile connected to GR on the elements 1.3 and 1.4 (Figure 1). A no less important concern for the patient was the dental hypersensitivity, associated with light pain during toothbrushing and during the assumption of cold or acidic food and drinks. Physical examination using a periodontal probe revealed the entity of the recessions and no reabsorption of interproximal bone peaks, confirmed by intraoral radiography. This led us to define the recession as Miller class I on both teeth (1.3, 1.4). The element 1.3 presented a recession of 3 mm from the gingival margin to the cementoenamel junction (CEJ) with a probing depth of 1 mm, while element 1.4 had a 2 mm recession with a probing depth of 1 mm. The tissues were not inflamed, and there were no signs of inconvenient muscular insertions. Given these considerations and once defined the diagnosis, we decided to perform a mucogingival surgery with bilaminar technique and CAF. We suggested to the patient the use of HAM in order to reduce postoperative morbidity and encouraging rapid healing without involving the palatal side. The HAM used in this case report was sourced, processed, and cryopreserved by “Fondazione Banca dei Tessuti di Treviso”, a tissue bank, as previously described by Martelloni et al. [14]. The surgical procedure started with the drawing and lifting of a partial thickness strip with two vertical drops, a distal one on 1.2 and a distal one on 1.5. The roots were scaled and conditioned using EDTA. The HAM graft was placed above the recession sites and stitched with a resorbable compressive suture (Figures 2–3) in order to allow adequate vascular flow for the integration and survival of the grafted tissue. The stitches were made with Vicryl 5.0 resorbable suture and with PTFE 6.0 nonresorbable suture (Figure 3). The HAM stromal side was placed on the periosteum, folded many times to gain the correct thickness, and then covered with the primary strip, adequately coronized, in a way to cover both the graft and the roots. The stitches were removed 15 days postintervention (Figure 4). The follow-up visits were performed every month for up to 7 months, a complete restoration of the gingival tissues and total absence of recession were registered (Figure 5). The bacterial plaque and the toothbrushing manoeuvres were monitored. At every visit, an increase in the thickness of the keratinized tissue (till 2 mm) was noticed as well as the maintenance of the natural colour of the gingiva without any form of inflammation (Figure 5).

## 3. Discussion

Mucogingival surgery techniques, currently used for covering Miller class I and II GR, are very predictable procedures in terms of results, both for bilaminar techniques and for mucogingival surgeries without the use of free grafts [6]. Previous articles demonstrated that harvesting autogenous free and connective tissue grafts from the palate result in higher patient morbidity, surgical time, and risk of complications [14]. Despite this, the withdrawal of connective tissue portion from the palate is necessary to thicken the keratinized fibrous tissue, cover the GRs, surround the dental implants and in fixed prosthesis on natural elements, or in order to modify the phenotype after orthodontic intervention on lower incisors with reduction of cortical vestibular. Every time it is necessary to retrieve the connective tissue from the palate, the surgeon asks himself how to avoid it, even if for different clinical scenarios autogenous grafts seems the best option. The encouraging and promising results, also in aesthetical terms, that we obtained with the use of HAM and the evidences reported in literature [15–17] led us assume that HAM is a valid alternative for treating the Miller class I and II GR. However, more studies using amniotic membrane for the treatment of gingival recession are needed. The mucogingival surgery with the use of HAM in a bilaminar technique with contextual CAF allows to treat the patient with success, reducing the biological cost of the intervention, obtaining a good functional and aesthetical result.

## 4. Conclusions

Within its limitation, the present study suggested that the use of bilaminar technique with CAF and HAM can be a valid option for treating Miller I and II gingival recessions.
